# Moving Beyond Disciplinary Silos Towards a Transdisciplinary Model of Wellbeing: An Invited Review

**DOI:** 10.3389/fpsyg.2021.642093

**Published:** 2021-05-14

**Authors:** Jessica Mead, Zoe Fisher, Andrew H. Kemp

**Affiliations:** ^1^Department of Psychology, College of Human and Health Sciences, Swansea University, Swansea, United Kingdom; ^2^Fieldbay, Swansea, United Kingdom; ^3^Health and Wellbeing Academy, College of Human and Health Sciences, Swansea University, Swansea, United Kingdom; ^4^Community Brain Injury Service, Morriston Hospital, Swansea, United Kingdom

**Keywords:** connection, emotion, GENIAL model, positive psychology, transdisciplinary science, wellbeing science

## Abstract

The construct of wellbeing has been criticised as a neoliberal construction of western individualism that ignores wider systemic issues such as inequality and anthropogenic climate change. Accordingly, there have been increasing calls for a broader conceptualisation of wellbeing. Here we impose an interpretative framework on previously published literature and theory, and present a theoretical framework that brings into focus the multifaceted determinants of wellbeing and their interactions across multiple domains and levels of scale. We define wellbeing as positive psychological experience, promoted by connections to self, community and environment, supported by healthy vagal function, all of which are impacted by socio-contextual factors that lie beyond the control of the individual. By emphasising the factors within and beyond the control of the individual and highlighting how vagal function both affects and are impacted by key domains, the biopsychosocial underpinnings of wellbeing are explicitly linked to a broader context that is consistent with, yet complementary to, multi-levelled ecological systems theory. Reflecting on the reciprocal relationships between multiple domains, levels of scale and related social contextual factors known to impact on wellbeing, our GENIAL framework may provide a foundation for a transdisciplinary science of wellbeing that has the potential to promote the wellbeing of individuals while also playing a key role in tackling major societal challenges.

## Introduction

Here we impose an interpretative framework on previously published literature and theory, laying a foundation for a transdisciplinary framework focused on better understanding and improving wellbeing. First, we briefly summarise some of the complexities and criticisms relating to wellbeing and its construct.

## Complexities and Criticisms of Wellbeing

The word “wellbeing” is not a simile for reduced illbeing, quality of life or happiness (Headey et al., 1985; Ryff et al., 2006; Westerhof and Keyes, 2010; Skevington and Böhnke, 2018). Our own work for example (Fisher et al., 2020; Tulip et al., 2020; Wilkie et al., 2021), has shown that wellbeing may be improved in neurological disorders, and in despite of significant levels of ill-health and distress. These findings reinforce Wong’s dual system model of what makes life worth living (Wong, 2012). Negative emotions provide the seeds for personal growth (Wong, 2010; Kashdan and Biswas-Diener, 2015), while major adversity and suffering can lead to “post-traumatic growth” (Joseph and Linley, 2006). Wellbeing interventions have been developed within disciplinary silos leading to a focus on isolated components [e.g., psychological interventions (Carr et al., 2020) are often distinct from the promotion of positive health behaviours (Buecker et al., 2020)]. The scientific focus on what constitutes a happy or good life has been described as “scientific polyannaism” (Yakushko, 2019), while the individual pursuit of wellbeing has been described as a socio-cultural construction of western individualism that places importance on wealth, fame and materialistic pursuits (Carlisle et al., 2009; Davies, 2015; Hull and Pasquale, 2018).

These complexities and criticisms highlight the need to transcend disciplinary boundaries and work towards a transdisciplinary model of wellbeing. Such an approach requires disciplinary integration and recontextualisation of competing theories in such a way that leads to new ideas and knowledge (Choi and Pak, 2006). Wellbeing must be conceptualised as a system, within which the interconnectedness of the individual in relation to their communities and environments must be explored while appreciating the impacts of socio-contextual factors (e.g., inequality, culture) that influence wellbeing and behaviour change theory to identify sustainable solutions for improving wellbeing. We further highlight a role for vagal nerve functioning, a psychophysiological index of wellbeing that affects and is affected by various determinants of wellbeing across multiple domains at multiple levels of scale, providing the theoretical glue around which our GENIAL framework has been developed (Kemp et al., 2017a; Mead et al., 2019; Fisher et al., 2020; Wilkie et al., 2021).

## Rethinking Wellbeing

Here we define wellbeing as positive psychological experience, promoted by connections to self, community and environment, supported by healthy vagal function, all of which are impacted by socio-contextual factors that lie beyond the control of the individual. Our manuscript has been structured around this definition, focusing on each of the domains within which wellbeing may arise, highlighting major socio-contextual factors that lie beyond the control of the individual, and discussing the capacity to sustain positive change, drawing on behaviour change theory at multiple levels of scale.

Self-connection is a relatively new concept, rooted in self-awareness that involves accepting and aligning behaviour based on that awareness (Klussman et al., 2020a,b,c). We suggest that “self-connection” may be supported by the vagus nerve, a structural link between body and mind. Self-connection is also associated with self-actualisation (Klussman et al., 2020a,b,c) and connectedness with others (Kok and Fredrickson, 2010; Kok et al., 2013), which has been described as a psychological need (Baumeister and Leary, 1995; Deci and Ryan, 2000). Social connectedness is associated with the social relational emotions including gratitude, compassion and awe, all of which are powerful determinants of prosocial behaviour (Stellar et al., 2017; Petersen et al., 2019). These emotions have been associated with higher levels of vagal function (Childre, 2004; Shiota et al., 2011; Bello et al., 2020), and recent thinking suggests that they may be involved in feelings of connection to the natural environment (Petersen et al., 2019) which are again associated with vagal nerve functioning (Richardson et al., 2016).

## The Vagus Nerve and Wellbeing

The vagus nerve connects the central nervous system to many different organs including heart, gut, liver and lungs. While the vagus nerve is one of several responses systems contributing to the experience of wellbeing, it has a regulatory role over many including the sympathetic nervous system (Porges, 2011; Deuchars et al., 2018), hypothalamic-pituitary adrenal axis (Porges, 2011), immune functioning (Tracey, 2002; Pavlov and Tracey, 2012), brain-gut interactions (Bonaz et al., 2018; Fülling et al., 2019) neurogenesis and epigenetic mechanisms (Follesa et al., 2007; Biggio et al., 2009; O’Leary et al., 2018). Research now links vagal function to positive emotions (Geisler et al., 2010; Kok and Fredrickson, 2010; Kok et al., 2013), meaning and purpose in life (Zilioli et al., 2015; Dang et al., 2021), emotion regulation (Geisler et al., 2010; Williams et al., 2015), executive function (Williams et al., 2019; Eggenberger et al., 2020), psychological flexibility (Kashdan and Rottenberg, 2010; Colzato et al., 2018), prosocial behaviours (Kemp et al., 2012; Geisler et al., 2013; Kok et al., 2013), positive health behaviours (Werner et al., 2015; Young and Benton, 2018), biopsychosocial resilience (Dedoncker et al., 2021), time spent in nature (Richardson et al., 2016; De Brito et al., 2020) and future morbidity and mortality (Hillebrand et al., 2013; Jandackova et al., 2016; Fang et al., 2020). Various theoretical models have been proposed within which these findings have been interpreted. The neurovisceral integration model (Thayer et al., 2009) presents the vagus nerve as a structural link between mind and body, arguably representing a psychophysiological correlate of self-connection. An iteration of this model (Kemp et al., 2017b) described HRV as a missing structural link between psychological moments and mortality, mediating the association between wellbeing and longevity vs. illbeing and premature mortality. Polyvagal theory (Porges, 2011) illustrates a role for the vagus nerve in the social engagement system, supporting the capacity for social connection (Holt-Lunstad et al., 2010; Kemp et al., 2017a). The evolutionary model for the wellbeing benefits of nature (Richardson et al., 2016) features the vagus nerve within a physiologically based model of affect. Interestingly, meta-analysis (Bello et al., 2020) has demonstrated a role for the vagus nerve in feelings of compassion, an experience supporting connection to self, others and nature (Neff, 2003; Petersen et al., 2019). Compassion is often facilitated through loving kindness meditation, which builds positive emotions, promotes feelings of social connectedness and raises levels of vagal function in an upward spiral relationship (Kok et al., 2013).

In summary, we view healthy vagal functioning as fundamental in supporting an individual’s capacity for connection to self, others and nature, while also acknowledging external impacts on vagal functioning that impact wellbeing.

## The Individual Domain and Wellbeing

Mental and physical wellbeing are core components of overall health that are intimately and bidirectionally associated (Kemp and Quintana, 2013; Steptoe et al., 2015). Mental wellbeing encompasses hedonic (positive emotions) and eudaimonic (flourishing) wellbeing (Diener et al., 1999; Ryan and Deci, 2001; Fredrickson, 2004; Wong, 2012; Ryff, 2014), and while competing theories have focused on one or the other, Seligman’s PERMA model—encompassing positive emotions, engagement, relationships, meaning and achievement—has characterised wellbeing as their combination (Seligman, 2012, 2017). Recent meta-analysis (Carr et al., 2020) reported that a variety of positive psychological interventions consistent with PERMA theory have small to medium effects on wellbeing (*g* = 0.39) as well as related outcome measures including character strengths (*g* = 0.46), quality of life (*g* = 0.48), depression (*g* = –0.39), anxiety (*g* = –0.62), and stress (*g* = –0.58). Findings from the English Longitudinal Study of Ageing reported that individuals with higher levels of eudaimonic wellbeing display a three-fold higher rate of survival over an 8.5-year follow-up period (Steptoe et al., 2015). Optimism is associated with a 11–15% longer lifespan and greater odds for achieving “exceptional longevity” (Lee et al., 2019). Vagal function may play a mediating role in these longevity outcomes (Zulfiqar et al., 2010; Kemp et al., 2017a, b; Hernández-Vicente et al., 2020).

The association between mental and physical wellbeing (*r* = 0.347) (Ngamaba et al., 2017) does not depend on whether objective or subjective measures of health status are used, or differ across those with or without chronic conditions (Ngamaba et al., 2017). More than 80% of the vagal nerve fibres are afferents, conveying sensory information from the viscera to the central nervous system (Yamakawa et al., 2015), providing an important communication pathway for the beneficial effects of positive health behaviours to influence brain and behaviour. As the vagus nerve provides a structural link between mind and body (Kemp et al., 2017b), we suggest that interventions focused on building mental and physical wellbeing may facilitate the experience of wellbeing to a greater extent than focusing on one or the other separately. As well as mental and physical wellbeing, the functioning of the vagus nerve is also associated with social connectedness (Kok and Fredrickson, 2010; Porges, 2011; Kok et al., 2013), the topic that we turn to next.

## The Community Domain and Wellbeing

There is much evidence to suggest that community is deteriorating (Kemp et al., 2017a), including generational shifts in narcissism (Twenge, 2013), declines in perspective taking and empathic concern (Konrath et al., 2011), increasing individualism (Santos et al., 2017) and inequality (Nolan and Valenzuela, 2019). Community is more than an aggregation of individuals, it is communicative and interactive, a dynamic process involving social interactions that support individual wellbeing (Brymer et al., 2020). Despite evidence of deterioration, humans are driven to connect with others, to feel a sense of attachment and belonging to the social group. This sense of relatedness with others is described as a basic psychological need (Baumeister and Leary, 1995; Deci and Ryan, 2000), and improvements in connectedness have been shown to improve public mental health (McNamara et al., 2013) year-on-year (Saeri et al., 2018). Individuals with stronger relationships have even been shown to have a 50% increased likelihood of survival over an average of 7.5 years follow-up (Holt-Lunstad et al., 2010). The theory of social wellbeing (Keyes, 1998, 2002; Westerhof and Keyes, 2010) is linked to the sense that society: is meaningful and understandable (social coherence); provides an opportunity for growth (social actualisation), is something that one belongs to and is accepted by (social acceptance and integration) and that one can meaningfully contribute to (social contribution). Accordingly, our focus extends beyond personal relationships, including concepts such as social capital, social cohesion and social identity. Social capital refers to connections between similar individuals (e.g., family and close friends; i.e., bonding social capital), people from diverse backgrounds (e.g., neighbours, members of sporting clubs, work colleagues; i.e., bridging social capital) and relationships characterised by power differences (e.g., the employee—employer relationship or that between citizen and government; i.e., linking social capital) (Putnam, 2000; Uphoff et al., 2013). Social capital protects against stress (Umberson and Montez, 2010) and is associated with positive emotions (Diener and Oishi, 2005) and wellbeing (Williams, 2006), especially in those with lower socioeconomic status (Uphoff et al., 2013). The related concept of social cohesion refers to the extent to which a geographical space achieves “community” through the sharing of values, cooperation and interaction (Beckley, 1995) Voluntary social participation promotes social cohesion in the community, creating a context for positive social relationships and eliciting feelings of belongingness and acceptance (De Vries et al., 2013; Elliott et al., 2014; Fonseca et al., 2018). Volunteering has been described as the “single most reliable way to momentarily increase ones well-being” (Seligman, 2012). Social identity theory provides a useful context for appreciating these effects on the wellbeing of individuals. Those who strongly identify with their community have display higher levels of wellbeing (McNamara et al., 2013). Social identity provides meaning and purpose to one’s life, facilitating feelings that one can collectively cope with the challenges. This sense of community is fostered through the promotion of social relational emotions, such as gratitude, compassion and awe, which may be linked to capacity for psychological connections to nature (Petersen et al., 2019), the topic that we turn to next.

## The Environment Domain and Wellbeing

Globalisation, urbanisation, and technological advancements has meant that humans have become increasingly disconnected from nature (Hartig et al., 2014; Chawla, 2015). This continues despite research showing that contact with nature improves wellbeing (Greenleaf et al., 2014; Capaldi et al., 2015; McMahan and Estes, 2015). Connection with nature contributes a small to medium effect to hedonic (*r* = 0.20) and eudaimonic (*r* = 0.24) wellbeing (Pritchard et al., 2019), and may reflect another fundamental psychological need (Richardson et al., 2020a). Researchers have even argued that one should consider spending up to 2 h per week in nature to experience wellbeing (White et al., 2019). Here in lies a conundrum: on the one hand, connection to the natural environment appears to be critically important for wellbeing, yet on the other, the impacts of climate change raises important ethical issues relating to focusing on the former, while ignoring the latter. It is interesting therefore to see emerging literature highlighting associations between nature connectedness and pro-environmental behaviours, in addition to wellbeing (Martin et al., 2020; Richardson et al., 2020b). Pro-environmental behaviours have been linked to psychological wellbeing (Verdugo, 2012) (Ganglmair-Wooliscroft and Wooliscroft, 2016; Venhoeven et al., 2016), positive emotion (O’Brien, 2008; Cloutier et al., 2014; Helliwell, 2017), and eudaimonic well-being (Venhoeven et al., 2013, 2016), social wellbeing (Prati et al., 2016) and community connectedness (Kweon et al., 1998). Furthermore, sustainability has been specifically linked to wellbeing, an idea that characterises the “positive psychology of sustainability” (Verdugo, 2012; Corral-Verdugo et al., 2015; Corral-Verdugo and Frías-Armenta, 2016), with researchers highlighting the positive psychological consequences of pro-ecological, altruistic, frugal and equitable behaviour (Corral-Verdugo et al., 2011, 2015). While our framework places the individual within the context of their social and natural ecologies, consistent with recent developments in wellbeing science (Lomas, 2015; Nielsen and Ma, 2018), there is also a need to consider factors that impact on wellbeing that lie well beyond the control of individuals. We turn our attention to such factors next.

## Socio-Contextual Factors and Wellbeing

A wide range of socio-contextual factors either facilitate or restrict the experience of wellbeing. Epidemiological studies demonstrate an association between proximity to green spaces and reductions in all-cause mortality including circulatory disease, ischemic stroke and depression (Mitchell and Popham, 2008; Wilker et al., 2014; Helbich et al., 2018). Yet, the advantages to health and wellbeing derived from proximity to green spaces are undermined by inequality with greater efforts needed to increase green space proximity for people of colour and lower income groups (Saporito and Casey, 2015). Inequality is perhaps one of the most discussed issues impacting on the wellbeing of populations. The most economically disadvantaged in society are disproportionally impacted by major societal challenges including increasing burden of chronic disease, societal loneliness and anthropogenic climate change (Cesare et al., 2013; Niedzwiedz et al., 2016; Otto et al., 2017). However, economic inequality also has adverse impacts on the entire population, contributing to multiple health and social problems, causally impacting on a variety of outcomes including educational attainment, obesity and homicide (Pickett and Wilkinson, 2015). Accordingly, improving economic inequality is fundamental to improving population wellbeing (Wilkinson and Pickett, 2010, 2019), and is a strategy featuring prominently in initiatives such as the Green New Deal (GND) (Galvin and Healy, 2020). Unlike narrow economic solutions, such as carbon taxes and emissions trading schemes, a GND would involve major societal and economic transformation driven by respect for human rights, social equity and societal wellbeing.

Culture is another important socio-contextual factor which has been shown to influence the way in which emotion and wellbeing is experienced and appraised (Diener and Suh, 2000; Ahuvia, 2002; Steptoe et al., 2007). While “individualistic” cultures prioritise positive emotions and personal wellbeing (Diener and Suh, 2000; Ahuvia, 2002; Steptoe et al., 2007), “collectivist” cultures place greater emphasis on emotional stability than on positive affect (Lu, 2001; Ng et al., 2003). Accordingly, wellbeing in individualistic cultures is more strongly associated with self-esteem and a sense of personal achievement, while wellbeing in collectivistic cultures is more strongly associated with avoiding social conflict and achieving interpersonal goals (Uchida and Oishi, 2016). Recent work has examined the impact of socio-contextual factors on vagal function (Kemp et al., 2016; Yang and Immordino-Yang, 2017) reporting that the vagus may support the capacity for emotional regulation associated with racial discrimination (Kemp et al., 2016) while other research has reported that healthy vagal functioning may predispose bicultural individuals to adopt a cultural identity that emphasises calmness (Yang and Immordino-Yang, 2017).

The discipline of psychology has focused mostly on western, educated, industrialised, rich and democratic (WEIRD) samples (Henrich et al., 2010), with as many as 78.2% of publications in positive psychology (up to 2018) associated with Western countries (Hendriks et al., 2018). While all people have fundamental needs including the need for happiness, meaning and self-determination, the expression and attainment of those universal values may be culture-bound (Wong, 2013). There is a growing appreciation for cultural differences in wellbeing, leading to new pluralistic measures (Lambert et al., 2020) that include a combination of hedonia, eudaimonia, social wellbeing, and the roles of culture, community, nature, and governance. We now turn our attention to the topic of how positive change might be sustained.

## Sustaining Positive Change

There is an inherent disconnect between what people know and what they actually do; known as the intention-behaviour gap (Sheeran, 2002). This represents a major barrier to translating evidence surrounding well-being activities into sustained practice (Francis et al., 2012). Emotions act as an important mediator in the intention–behaviour gap and emotionally based interventions may increase the efficacy of behaviour change interventions (Mohiyeddini et al., 2009). According to the upward spiral model of lifestyle change (Cappellen et al., 2017), positive affect experienced during health behaviours increases non-conscious motives for those behaviours, while vagal nerve functioning provides a biological resource for positive change. A review of 100 behaviour change theories identified five overarching, interconnected themes relating to effective behavioural change strategies (Kwasnicka et al., 2016). Themes reflected the differential nature and role of motives, self-regulation, habits, psychological and physical resources, and environmental and social influences from initiation to maintenance. Subtle behavioural “nudging” has also been shown to successfully change behaviours at the societal level (Gill and Boylan, 2012; Marteau et al., 2012). However, nudging is underpinned by “libertarian paternalism,” which walks a fine line between upholding individual freedom and subliminal state manipulation of the “cognitively crippled.” An alternative approach involves “psychological boosting,” guided by a much more positive view of humanity described as “ecological rationality” in which non-rationality is viewed as an adaptive capacity to be valued (Gigerenzer, 2018; Hertwig et al., 2019; Fabian and Pykett, 2021). This approach develops capacity, empowerment and participation of individuals and may help in promoting societal and economic transformation through wellbeing public policy (Fabian and Pykett, 2021). Metrics such as the Happy Planet Index^1^ now rank countries on the basis of a combination of wellbeing (life satisfaction), life expectancy, inequality of outcomes and ecological footprint, facilitating conversations, and driving actions to achieving sustainable development goals while promoting wellbeing of individuals as well as the communities and environments within which people live (Patrick et al., 2019). These metrics may help to facilitate a shift in focus from GDP to wellbeing as has been done in New Zealand, Iceland, Scotland, and Wales, the “so-called” economies of wellbeing (Fabian and Pykett, 2021).

## Discussion and Conclusion

The framework we present emphasises core inter-related domains that span the individual, community and environment, encompassing major determinants of wellbeing. Our framework has also been inspired by and builds on recent developments (Kemp et al., 2017a; Kemp, 2019; Kern et al., 2019; Mead et al., 2019; Wong, 2019; Lomas et al., 2020), characterised as second and third wave positive psychology (Wong, 2019; Lomas et al., 2020), which place importance on emotional balance, meaning and purpose, interconnectedness and interdisciplinarity. The framework presented here has already contributed to a better understanding of how to protect wellbeing during the COVID pandemic (Mead et al., 2020) and has led to the development of an innovative wellbeing science intervention, targeting a variety of populations including university students (Kemp et al., 2021) and people living with neurological disorders (Fisher et al., 2020), with a focus on acquired brain injury (Tulip et al., 2020; Wilkie et al., 2021).

By emphasising the inter-connectedness across domains and levels of scale, our framework encourages thinking about how to promote wellbeing while simultaneously ameliorating major societal challenges. Take for example, the challenge of climate change in which behavioural and lifestyle choices together with larger collaborative efforts will be essential for successful adaptation (IPCC, 2014). At an individual level, nature connection can be enhanced through nature-enhanced meditation (Ray et al., 2020), gardening (Blair et al., 1991; Okvat and Zautra, 2011), and physical exercise (Coon et al., 2011). At a community level, peaceful environmental activism (Klar and Kasser, 2009) and volunteering (Binder and Freytag, 2013; Binder and Blankenberg, 2016) offer ways to increase subjective wellbeing, community connectedness while promoting pro-environmental behaviours (Jackson, 2005; Okvat and Zautra, 2011; Ibáñez-Rueda et al., 2020). In the clinical setting “green care” interventions, such as care farming, horticultural therapy, wilderness therapy, ecotherapy, sustainable building etc., have been shown to improve wellbeing (Haubenhofer et al., 2010; Whear et al., 2014; Wright and Wadsworth, 2014; Wendelboe-Nelson et al., 2019; Fisher et al., 2020; Tulip et al., 2020; Wilkie et al., 2021). Environmental modifications such as the commissioning of outdoor gym equipment (Cranney et al., 2016), provision of community gardens (Veitch et al., 2012), walking or bike trials and improved accessibility of parks or gardens (Fraser and Lock, 2011) have the potential to increase nature connectedness, pro-sustainable behaviours and positive health behaviours (diet and physical activity), contributing to improved population health and wellbeing (see (Shanahan et al., 2019) for a review). Corporate sustainability strategies have considerable scope to improve environmental outcomes, especially when employees are involved in the development of these strategies, while global initiatives such as the GND are needed to facilitate societal transformation (Boiral, 2005; Michailides and Lipsett, 2013). Finally, any initiatives must specifically consider socio-cultural values, which determine the way in which people use natural resources, the extent of their pro-environmental behaviours (Ringov and Zollo, 2007) as well as specific determinants of wellbeing (Diener and Suh, 2000; Ahuvia, 2002; Steptoe et al., 2007).

In conclusion, focusing on wellbeing across multiple domains at increasingly higher levels of scale offers underrealised potential to ameliorate social challenges, while also promoting wellbeing of individuals. The framework presented here may provide a foundation for thinking about how this might be achieved, while working towards a transdisciplinary science of wellbeing.

## Author Contributions

JM wrote the first iteration of this manuscript. All authors refined and further developed the manuscript for publication.

## Conflict of Interest

We acknowledge funding that we have received from our industry partner, Fieldbay, which has been used to co-fund a Ph.D. studentship that was awarded to JM. The remaining authors declare that the research was conducted in the absence of any commercial or financial relationships that could be construed as a potential conflict of interest.

## Footnotes

1 http://happyplanetindex.org/

## References

[B1] Ahuvia A. C. (2002). Individualism/collectivism and cultures of happiness: a theoretical conjecture on the relationship between consumption, culture and subjective well-being at the national level. *J. Happiness Stud.* 3 23–36.

[B2] Baumeister R. F. Leary M. R. (1995). The need to belong: desire for interpersonal attachments as a fundamental human motivation. *Psychol. Bull.* 117 497–529. 10.1037/0033-2909.117.3.497 7777651

[B3] Beckley T. M. (1995). Community stability and the relationship between economic and social well-being in forest-dependent communities. *Soc. Nat. Resour.* 8 261–266. 10.1080/08941929509380919

[B4] Bello M. D. Carnevali L. Petrocchi N. Thayer J. F. Gilbert P. Ottaviani C. (2020). The compassionate vagus: a meta-analysis on the connection between compassion and heart rate variability. *Neurosci. Biobehav. Rev.* 116 21–30. 10.1016/j.neubiorev.2020.06.016 32554001

[B5] Biggio F. Gorini G. Utzeri C. Olla P. Marrosu F. Mocchetti I. (2009). Chronic vagus nerve stimulation induces neuronal plasticity in the rat hippocampus. *Int. J. Neuropsychopharmacol.* 12:1209. 10.1017/s1461145709000200 19309534 PMC2879889

[B6] Binder M. Blankenberg A.-K. (2016). Environmental concerns, volunteering and subjective well-being: antecedents and outcomes of environmental activism in Germany. *Ecol. Econ.* 124 1–16. 10.1016/j.ecolecon.2016.01.009

[B7] Binder M. Freytag A. (2013). Volunteering, subjective well-being and public policy. *J. Econ. Psychol.* 34 97–119. 10.1016/j.joep.2012.11.008

[B8] Blair D. Giesecke C. C. Sherman S. (1991). A dietary, social and economic evaluation of the Philadelphia urban gardening project. *J. Nutr. Educ.* 23 161–167. 10.1016/s0022-3182(12)81191-5

[B9] Boiral O. (2005). The impact of operator involvement in pollution reduction: case studies in Canadian chemical companies. *Bus. Strateg. Environ.* 14 339–360. 10.1002/bse.431

[B10] Bonaz B. Bazin T. Pellissier S. (2018). The vagus nerve at the interface of the microbiota-gut-brain axis. *Front. Neurosci.* 12:49. 10.3389/fnins.2018.00049 29467611 PMC5808284

[B11] Brymer A. L. B. Toledo D. Spiegal S. Pierson F. Clark P. E. Wulfhorst J. D. (2020). Social-ecological processes and impacts affect individual and social well-being in a rural western U.S. landscape. *Front. Sustain Food Syst.* 4:38. 10.3389/fsufs.2020.00038

[B12] Buecker S. Simacek T. Ingwersen B. Terwiel S. Simonsmeier B. A. (2020). Physical activity and subjective well-being in healthy individuals: a meta-analytic review. *Health Psychol. Rev.* 1–19. 10.1080/17437199.2020.1760728 32452716

[B13] Capaldi C. A. Passmore H.-A. Nisbet E. K. Zelenski J. M. Dopko R. L. (2015). Flourishing in nature: a review of the benefits of connecting with nature and its application as a wellbeing intervention. *Int. J. Wellbeing* 5 1–16. 10.5502/ijw.v5i4.1 32285354

[B14] Cappellen P. V. Rice E. L. Catalino L. I. Fredrickson B. L. (2017). Positive affective processes underlie positive health behaviour change. *Psychol. Health* 33 1–21. 10.1080/08870446.2017.1320798 PMC5682236 28498722

[B15] Carlisle S. Henderson G. Hanlon P. W. (2009). ‘Wellbeing’: a collateral casualty of modernity? *Soc. Sci. Med.* 69 1556–1560. 10.1016/j.socscimed.2009.08.029 19765875

[B16] Carr A. Cullen K. Keeney C. Canning C. Mooney O. Chinseallaigh E. (2020). Effectiveness of positive psychology interventions: a systematic review and meta-analysis. *J. Posit. Psychol.* 1–21. 10.1080/17439760.2020.1818807

[B17] Cesare M. D. Khang Y.-H. Asaria P. Blakely T. Cowan M. J. Farzadfar F. (2013). Inequalities in non-communicable diseases and effective responses. *Lancet* 381 585–597. 23410608 10.1016/S0140-6736(12)61851-0

[B18] Chawla L. (2015). Benefits of nature contact for children. *J. Plan. Lit.* 30 433–452. 10.1177/0885412215595441

[B19] Childre R. M. D. (2004). *The Psychology of Gratitude.* Oxford: Oxford University Press, 230–256.

[B20] Choi B. Pak A. (2006). Multidisciplinarity, interdisciplinarity and transdisciplinarity in health research, services, education and policy: 1. Definitions, objectives, and evidence of effectiveness. *Clin. Invest. Med.* 29 351–364. 17330451

[B21] Cloutier S. Larson L. Jambeck J. (2014). Are sustainable cities “happy” cities? Associations between sustainable development and human well-being in urban areas of the United States. *Environ. Dev. Sustain.* 16 633–647. 10.1007/s10668-013-9499-0

[B22] Colzato L. S. Jongkees B. J. de Wit M. van der Molen M. J. W. Steenbergen L. (2018). Variable heart rate and a flexible mind: higher resting-state heart rate variability predicts better task-switching. *Cogn. Affect. Behav. Neurosci.* 18 730–738. 10.3758/s13415-018-0600-x 29713957 PMC6096636

[B23] Coon J. T. Boddy K. Stein K. Whear R. Barton J. Depledge M. H. (2011). Does participating in physical activity in outdoor natural environments have a greater effect on physical and mental wellbeing than physical activity indoors? A systematic review. *Environ. Sci. Technol.* 45 1761–1772. 10.1021/es102947t 21291246

[B24] Corral-Verdugo V. Frías-Armenta M. (2016). The sustainability of positive environments. *Environ. Dev. Sustain.* 18 965–984. 10.1007/s10668-015-9701-7

[B25] Corral-Verdugo V. Mireles-Acosta J. Tapia-Fonllem C. Fraijo-Sing B. (2011). Happiness as correlate of sustainable behavior. *Hum. Ecol. Rev.* 18 95–104.

[B26] Corral-Verdugo V. Tapia-Fonllem C. Ortiz-Valdez A. (2015). On the relationship between character strengths and sustainable behavior. *Environ. Behav.* 47 877–901. 10.1177/0013916514530718

[B27] Cranney L. Phongsavan P. Kariuki M. Stride V. Scott A. Hua M. (2016). Impact of an outdoor gym on park users’ physical activity: a natural experiment. *Health Place* 37 26–34. 10.1016/j.healthplace.2015.11.002 26699448

[B28] Dang K. Kirk M. A. Monette G. Katz J. Ritvo P. (2021). Meaning in life and vagally-mediated heart rate variability: evidence of a quadratic relationship at baseline and vagal reactivity differences. *Int. J. Psychophysiol.* 165 101–111. 33745963 10.1016/j.ijpsycho.2021.03.001

[B29] Davies W. (2015). *The Happiness Industry.* Brooklyn, NY: Verso Books.

[B30] De Brito J. N. Pope Z. C. Mitchell N. R. Schneider I. E. Larson J. M. Horton T. H. (2020). The effect of green walking on heart rate variability: a pilot crossover study. *Environ. Res.* 185:109408. 10.1016/j.envres.2020.109408 32220745 PMC7877549

[B31] De Vries S. van Dillen S. M. E. Groenewegen P. P. Spreeuwenberg P. (2013). Streetscape greenery and health: stress, social cohesion and physical activity as mediators. *Soc. Sci. Med.* 94 26–33. 10.1016/j.socscimed.2013.06.030 23931942

[B32] Deci E. L. Ryan R. M. (2000). The “what” and “why” of goal pursuits: human needs and the self-determination of behavior. *Psychol. Inq.* 11 227–268. 10.1207/s15327965pli1104_01

[B33] Dedoncker J. Vanderhasselt M.-A. Ottaviani C. Slavich G. M. (2021). Mental health during the covid-19 pandemic and beyond: the importance of the vagus nerve for biopsychosocial resilience. *Neurosci. Biobehav. Rev.* 125 1–10. 10.1016/j.neubiorev.2021.02.010 33582230 PMC8106638

[B34] Deuchars S. A. Lall V. K. Clancy J. Mahadi M. Murray A. Peers L. (2018). Mechanisms underpinning sympathetic nervous activity and its modulation using transcutaneous vagus nerve stimulation. *Exp. Physiol.* 103 326–331. 10.1113/ep086433 29205954 PMC5887928

[B35] Diener E. Oishi S. (2005). The nonobvious social psychology of happiness. *Psychol. Inq.* 16 162–167. 10.1207/s15327965pli1604_04

[B36] Diener E. Suh E. M. (2000). *Culture and Subjective Well-Being.* London: MIT press.

[B37] Diener E. Suh E. M. Lucas R. E. Smith H. L. (1999). Subjective well-being: three decades of progress. *Psychol. Bull.* 125 276–302. 10.1037/0033-2909.125.2.276

[B38] Eggenberger P. Annaheim S. Kündig K. A. Rossi R. M. Münzer T. de Bruin E. D. (2020). Heart rate variability mainly relates to cognitive executive functions and improves through exergame training in older adults: a secondary analysis of a 6-month randomized controlled trial. *Front. Aging Neurosci.* 12:197. 10.3389/fnagi.2020.00197 32760267 PMC7373948

[B39] Elliott J. Gale C. R. Parsons S. Kuh D. (2014). Neighbourhood cohesion and mental wellbeing among older adults: a mixed methods approach. *Soc. Sci. Med.* 107 44–51. 10.1016/j.socscimed.2014.02.027 24602970

[B40] Fabian M. Pykett J. (2021). Be happy: navigating normative issues in behavioral and well-being public policy. *Perspect. Psychol. Sci.* 10.1177/1745691620984395 [Epub ahead of print]. 33682526 PMC8785257

[B41] Fang S.-C. Wu Y.-L. Tsai P.-S. (2020). Heart rate variability and risk of all-cause death and cardiovascular events in patients with cardiovascular disease: a meta-analysis of cohort studies. *Biol. Res. Nurs.* 22 45–56. 10.1177/1099800419877442 31558032

[B42] Fisher Z. Galloghly E. Boglo E. Gracey F. Kemp A. H. (2020). “Emotion, wellbeing and the neurological disorders,” in *Reference Module in Neuroscience and Biobehavioral Psychology* (Amsterdam: Elsevier).

[B43] Follesa P. Biggio F. Gorini G. Caria S. Talani G. Dazzi L. (2007). Vagus nerve stimulation increases norepinephrine concentration and the gene expression of BDNF and bFGF in the rat brain. *Brain Res.* 1179 28–34. 10.1016/j.brainres.2007.08.045 17920573

[B44] Fonseca X. Lukosch S. Brazier F. (2018). Social cohesion revisited: a new definition and how to characterize it. *Innov. Eur. J. Soc. Sci. Res.* 32 231–253. 10.1080/13511610.2018.1497480

[B45] Francis J. J. O’Connor D. Curran J. (2012). Theories of behaviour change synthesised into a set of theoretical groupings: introducing a thematic series on the theoretical domains framework. *Implement. Sci.* 7:35. 22531601 10.1186/1748-5908-7-35 PMC3444902

[B46] Fraser S. D. S. Lock K. (2011). Cycling for transport and public health: a systematic review of the effect of the environment on cycling. *Eur. J. Public Health* 21 738–743. 10.1093/eurpub/ckq145 20929903

[B47] Fredrickson B. L. (2004). The broaden-and-build theory of positive emotions. *Philos. Trans. R. Soc. Lond. Ser. B Biol. Sci.* 359:1367–1377. 15347528 10.1098/rstb.2004.1512 PMC1693418

[B48] Fülling C. Dinan T. G. Cryan J. F. (2019). Gut microbe to brain signaling: what happens in vagus…. *Neuron* 101 998–1002. 10.1016/j.neuron.2019.02.008 30897366

[B49] Galvin R. Healy N. (2020). The green new deal in the United States: what it is and how to pay for it. *Energy Res. Soc. Sci.* 67:101529. 10.1016/j.erss.2020.101529

[B50] Ganglmair-Wooliscroft A. Wooliscroft B. (2016). Diffusion of innovation: the case of ethical tourism behavior. *J. Bus. Res.* 69 2711–2720. 10.1016/j.jbusres.2015.11.006

[B51] Geisler F. C. M. Kubiak T. Siewert K. Weber H. (2013). Cardiac vagal tone is associated with social engagement and self-regulation. *Biol. Psychol.* 93 279–286. 10.1016/j.biopsycho.2013.02.013 23466587

[B52] Geisler F. C. M. Vennewald N. Kubiak T. Weber H. (2010). The impact of heart rate variability on subjective well-being is mediated by emotion regulation. *Pers. Indiv. Differ.* 49:723728. 10.1016/j.paid.2010.06.015

[B53] Gigerenzer G. (2018). The bias bias in behavioral economics. *Rev. Behav. Econ.* 5 303–336. 10.1561/105.00000092

[B54] Gill T. P. Boylan S. (2012). Public health messages: why are they ineffective and what can be done? *Curr. Obes. Rep.* 1 50–58. 10.1007/s13679-011-0003-6

[B55] Greenleaf A. T. Bryant R. M. Pollock J. B. (2014). Nature-based counseling: integrating the healing benefits of nature into practice. *Int. J. Adv. Couns.* 36 162–174. 10.1007/s10447-013-9198-4

[B56] Hartig T. Mitchell R. de Vries S. Frumkin H. (2014). Nature and health. *Annu. Rev. Publ. Health* 35 207–228. 10.1146/annurev-publhealth-032013-182443 24387090

[B57] Haubenhofer D. K. Elings M. Hassink J. Hine R. E. (2010). The development of green care in western European countries. *Explor. J. Sci. Heal.* 6 106–111. 10.1016/j.explore.2009.12.002 20362268

[B58] Headey B. Holmstrom E. Wearing A. (1985). Models of well-being and ill-being. *Soc. Indic. Res.* 17 211–234. 10.1007/bf00319311

[B59] Helbich M. Klein N. Roberts H. Hagedoorn P. Groenewegen P. P. (2018). More green space is related to less antidepressant prescription rates in the Netherlands: a bayesian geoadditive quantile regression approach. *Environ. Res.* 166 290–297. 10.1016/j.envres.2018.06.010 29936285

[B60] Helliwell J. F. (2017). Social norms, happiness, and the environment: closing the circle. *Sustain. Sci. Pract. Policy* 10 78–84. 10.1080/15487733.2014.11908126

[B61] Hendriks T. Warren M. A. Schotanus-Dijkstra M. Hassankhan A. Graafsma T. Bohlmeijer E. (2018). How WEIRD are positive psychology interventions? A bibliometric analysis of randomized controlled trials on the science of well-being. *J. Posit. Psychol.* 14 489–501. 10.1080/17439760.2018.1484941

[B62] Henrich J. Heine S. J. Norenzayan A. (2010). The weirdest people in the world? *Behav. Brain Sci.* 33 61–83; discussion 83–135. 20550733 10.1017/S0140525X0999152X

[B63] Hernández-Vicente A. Hernando D. Santos-Lozano A. Rodríguez-Romo G. Vicente-Rodríguez G. Pueyo E. (2020). Heart rate variability and exceptional longevity. *Front Physiol.* 11:566399. 10.3389/fphys.2020.566399 33041862 PMC7527628

[B64] Hertwig R. Pleskac T. J. Centre for Adaptive Rationality Pachur T. (2019). *Taming Uncertainty.* Cambridge, MA: MIT Press.

[B65] Hillebrand S. Gast K. B. de Mutsert R. Swenne C. A. Jukema J. W. Middeldorp S. (2013). Heart rate variability and first cardiovascular event in populations without known cardiovascular disease: meta-analysis and dose–response meta-regression. *Europace* 15 742–749. 10.1093/europace/eus341 23370966

[B66] Holt-Lunstad J. Smith T. B. Layton J. B. (2010). Social relationships and mortality risk: a meta-analytic review. *PLoS Med.* 7:e1000316. 10.1371/journal.pmed.1000316 20668659 PMC2910600

[B67] Hull G. Pasquale F. (2018). Toward a critical theory of corporate wellness. *Biosocieties* 13 190–212. 10.1057/s41292-017-0064-1

[B68] Ibáñez-Rueda N. Guillén-Royo M. Guardiola J. (2020). Pro-environmental behavior, connectedness to nature, and wellbeing dimensions among granada students. *Sustainability* 12:9171. 10.3390/su12219171

[B69] IPCC (2014). “Climate change 2014: mitigation of climate change,” in *Contribution of Working Group III to the Fifth Assessment Report of the IPCC*, (Cambridge: Cambridge University Press).

[B70] Jackson T. (2005). Live better by consuming less? Is there a ‘double dividend’ in sustainable consumption? *J. Ind. Ecol.* 9 19–36. 10.1162/1088198054084734

[B71] Jandackova V. K. Britton A. Malik M. Steptoe A. (2016). Heart rate variability and depressive symptoms: a cross-lagged analysis over a 10-year period in the Whitehall II study. *Psychol. Med.* 46 1–11. 27181276 10.1017/S003329171600060X

[B72] Jandackova V. K. Scholes S. Britton A. Steptoe A. (2019). Healthy lifestyle and cardiac vagal modulation over 10 years: Whitehall II cohort study. *J. Am. Heart Assoc.* 8:e012420. 31547790 10.1161/JAHA.119.012420 PMC6806037

[B73] Joseph S. Linley P. A. (2006). Growth following adversity: theoretical perspectives and implications for clinical practice. *Clin. Psychol. Rev.* 26 1041–1053. 10.1016/j.cpr.2005.12.006 16473442

[B74] Kashdan T. B. Rottenberg J. (2010). Psychological flexibility as a fundamental aspect of health. *Clin. Psychol. Rev.* 30 865–878. 10.1016/j.cpr.2010.03.001 21151705 PMC2998793

[B75] Kashdan T. Biswas-Diener R. (2015). *The Upside of Your Dark Side: Why Being Your Whole Self–Not Just Your “Good” Self–Drives Success and Fulfillment.* New York, NY: Plume Books.

[B76] Kemp A. H. (2019). *Toward a Transdisciplinary Science of Health and Wellbeing Spanning Psychological Science and Epidemiology: A Focus on Vagal Function.* Melbourne, VIC: University of Melbourne.

[B77] Kemp A. H. Quintana D. S. (2013). The relationship between mental and physical health: insights from the study of heart rate variability. *Int. J. Psychophysiol.* 89 288–296. 10.1016/j.ijpsycho.2013.06.018 23797149

[B78] Kemp A. H. Arias J. A. Fisher Z. (2017a). “Social ties, health and wellbeing: a literature review and model,” in *Neuroscience and Social Science*, Vol. 59 eds Ibáñez A. Sedeño L. García A. (Cham: Springer International Publishing), 397–427. 10.1007/978-3-319-68421-5_17

[B79] Kemp A. H. Koenig J. Thayer J. (2017b). From psychological moments to mortality: a multidisciplinary synthesis on heart rate variability spanning the continuum of time. *Neurosci. Biobehav. Rev.* 83:567. 10.1016/j.neubiorev.2017.09.006 28888535

[B80] Kemp A. H. Koenig J. Thayer J. F. Bittencourt M. S. Pereira A. C. Santos I. S. (2016). Race and resting-state heart rate variability in brazilian civil servants and the mediating effects of discrimination. *Psychosom. Med.* 78 950–958. 10.1097/psy.0000000000000359 27359180

[B81] Kemp A. H. Mead J. Sandhu S. Fisher Z. (2021). Teaching wellbeing science. *Open Sci. Framework* 10.17605/OSF.IO/E7ZJF

[B82] Kemp A. H. Quintana D. S. Kuhnert R.-L. Griffiths K. Hickie I. B. Guastella A. J. (2012). Oxytocin increases heart rate variability in humans at rest: implications for social approach-related motivation and capacity for social engagement. *PLoS One* 7:e44014. 10.1371/journal.pone.0044014 22937145 PMC3429409

[B83] Kern M. L. Williams P. Spong C. Colla R. Sharma K. Downie A. (2019). Systems informed positive psychology. *J. Posit. Psychol.* 15 1–11. 10.1017/cbo9781139857109.002

[B84] Keyes C. L. M. (1998). Social well-being. *Soc. Psychol. Q.* 61:121.

[B85] Keyes C. L. M. (2002). The mental health continuum: from languishing to flourishing in life. *J. Health Soc. Behav.* 43:207. 10.2307/3090197 12096700

[B86] Klar M. Kasser T. (2009). Some benefits of being an activist: measuring activism and its role in psychological well-being. *Polit. Psychol.* 30 755–777. 10.1111/j.1467-9221.2009.00724.x

[B87] Klussman K. Curtin N. Langer J. Nichols A. L. (2020a). Examining the effect of mindfulness on well-being: self-connection as a mediator. *J. Pac. Rim Psychol.* 14:e5. 10.1017/prp.2019.29

[B88] Klussman K. Langer J. Nichols A. L. Curtin N. (2020b). What’s stopping Us from connecting with ourselves? A qualitative examination of barriers to self-connection. *Int. J. Appl. Posit. Psychol..* 5 137–152. 10.1007/s41042-020-00031-x

[B89] Klussman K. Nichols A. L. Langer J. (2020c). The role of self-connection in the relationship between mindfulness and meaning: a longitudinal examination. *Appl. Psychol. Heal Well* 12 636–659. 10.1111/aphw.12200 32333526

[B90] Kok B. E. Fredrickson B. L. (2010). Upward spirals of the heart: autonomic flexibility, as indexed by vagal tone, reciprocally and prospectively predicts positive emotions and social connectedness. *Biol. Psychol.* 85 432–436. 10.1016/j.biopsycho.2010.09.005 20851735 PMC3122270

[B91] Kok B. E. Coffey K. A. Cohn M. A. Catalino L. I. Vacharkulksemsuk T. Algoe S. B. (2013). How positive emotions build physical health. *Psychol. Sci.* 24 1123–1132. 10.1177/0956797612470827 23649562

[B92] Konrath S. H. O’Brien E. H. Hsing C. (2011). Changes in dispositional empathy in American college students over time: a meta-analysis. *Pers. Soc. Psychol. Rev.* 15 180–198. 10.1177/1088868310377395 20688954

[B93] Kwasnicka D. Dombrowski S. U. White M. Sniehotta F. (2016). Theoretical explanations for maintenance of behaviour change: a systematic review of behaviour theories. *Health Psychol. Rev.* 10 1–20. 26854092 10.1080/17437199.2016.1151372 PMC4975085

[B94] Kweon B.-S. Sullivan W. C. Wiley A. R. (1998). Green common spaces and the social integration of inner-city older adults. *Environ. Behav.* 30 832–858. 10.1177/001391659803000605

[B95] Lambert L. Lomas T. van de Weijer M. P. Passmore H. A. Joshanloo M. Harter J. (2020). Towards a greater global understanding of wellbeing: a proposal for a more inclusive measure. *Int. J. Wellbeing* 10 1–18. 10.5502/ijw.v10i2.1037 32285354

[B96] Lee L. O. James P. Zevon E. S. Kim E. S. Trudel-Fitzgerald C. Spiro A. (2019). Optimism is associated with exceptional longevity in 2 epidemiologic cohorts of men and women. *Proc. Natl. Acad. Sci. U.S.A.* 116 18357–18362. 10.1073/pnas.1900712116 31451635 PMC6744861

[B97] Lomas T. (2015). Positive social psychology: a multilevel inquiry into sociocultural well-being initiatives. *Psychol. Public Policy Law* 21 338–347. 10.1037/law0000051

[B98] Lomas T. Waters L. Williams P. Oades L. G. Kern M. L. (2020). Third wave positive psychology: broadening towards complexity. *J. Posit. Psychol.* 1–15. 10.1080/17439760.2020.1805501 [Epub ahead of print].

[B99] Lu L. (2001). Understanding happiness: a look into the Chinese folk psychology. *J. Happiness Stud.* 2 407–432.

[B100] Marteau T. M. Hollands G. J. Fletcher P. C. (2012). Changing human behavior to prevent disease: the importance of targeting automatic processes. *Science* 337 1492–1495. 10.1126/science.1226918 22997327

[B101] Martin L. White M. P. Hunt A. Richardson M. Pahl S. Burt J. (2020). Nature contact, nature connectedness and associations with health, wellbeing and pro-environmental behaviours. *J. Environ. Psychol.* 68:101389. 10.1016/j.jenvp.2020.101389

[B102] McMahan E. A. Estes D. (2015). The effect of contact with natural environments on positive and negative affect: a meta-analysis. *J. Posit. Psychol.* 10 507–519. 10.1080/17439760.2014.994224

[B103] McNamara N. Stevenson C. Muldoon O. T. (2013). Community identity as resource and context: a mixed method investigation of coping and collective action in a disadvantaged community. *Eur. J. Soc. Psychol.* 43 393–403. 10.1002/ejsp.1953

[B104] Mead J. Fisher Z. Tree J. Wong P. Kemp A. H. (2020). Predictors of wellbeing during the COVID-19 pandemic: key roles for gratitude and tragic optimism in a UK-based cohort. *PsyArXiv [Preprint]* 10.31234/osf.io/z2pxg PMC8295471 34305717

[B105] Mead J. Fisher Z. Wilkie L. Gibbs K. Pridmore J. Tree J. (2019). Toward a more ethical science of wellbeing that considers current and future generations. *Authorea* 10.22541/au.156649190.08734276 [Epub ahead of print].

[B106] Michailides T. P. Lipsett M. G. (2013). Surveying employee attitudes on corporate social responsibility at the frontline level of an energy transportation company. *Corp. Soc. Responsib. Environ. Manag.* 20 296–320. 10.1002/csr.1291

[B107] Mitchell R. Popham F. (2008). Effect of exposure to natural environment on health inequalities: an observational population study. *Lancet* 372 1655–1660. 10.1016/s0140-6736(08)61689-x 18994663

[B108] Mohiyeddini C. Pauli R. Bauer S. (2009). The role of emotion in bridging the intention–behaviour gap: the case of sports participation. *Psychol. Sport Exerc.* 10 226–234. 10.1016/j.psychsport.2008.08.005

[B109] Natarajan A. Pantelopoulos A. Emir-Farinas H. Natarajan P. (2020). Heart rate variability with photoplethysmography in 8 million individuals: a cross-sectional study. *Lancet Digital Heal.* 2 e650–e657. 10.1016/S2589-7500(20)30246-6 33328029

[B110] Neff K. (2003). Self-compassion: an alternative conceptualization of a healthy attitude toward oneself. *Self Identity* 2 85–101. 10.1080/15298860309032

[B111] Ng A. K. Ho D. Y. F. Wong S. S. Smith I. (2003). In search of the good life: a cultural odyssey in the east and west. *Genet. Soc. Gen. Psychol. Monogr.* 129 317–363. 15332721

[B112] Ngamaba K. H. Panagioti M. Armitage C. J. (2017). How strongly related are health status and subjective well-being? Systematic review and meta-analysis. *Eur. J. Public Health* 27 879–885. 10.1093/eurpub/ckx081 28957478

[B113] Niedzwiedz C. L. Richardson E. A. Tunstall H. Shortt N. K. Mitchell R. J. Pearce J. R. (2016). The relationship between wealth and loneliness among older people across Europe: is social participation protective? *Prev. Med.* 91 24–31. 10.1016/j.ypmed.2016.07.016 27471027

[B114] Nielsen T. W. Ma J. S. (2018). Connecting social and natural ecologies through a curriculum of giving for student wellbeing and engagement. *Aust. J. Environ. Educ.* 34 215–227. 10.1017/aee.2018.41

[B115] Nolan B. Valenzuela L. (2019). Inequality and its discontents. *Oxford Rev. Econ. Pol.* 35 396–430.

[B116] O’Brien C. (2008). Sustainable happiness: how happiness studies can contribute to a more sustainable future. *Can. Psychol.* 49 289–295. 10.1037/a0013235

[B117] O’Leary O. F. Ogbonnaya E. S. Felice D. Levone B. R. Conroy L. C. Fitzgerald P. (2018). The vagus nerve modulates BDNF expression and neurogenesis in the hippocampus. *Eur. Neuropsychopharmacol.* 28 307–316. 10.1016/j.euroneuro.2017.12.004 29426666

[B118] Okvat H. A. Zautra A. J. (2011). Community gardening: a parsimonious path to individual, community, and environmental resilience. *Am. J. Commun. Psychol.* 47 374–387. 10.1007/s10464-010-9404-z 21222153

[B119] Otto I. M. Reckien D. Reyer C. P. O. Marcus R. Masson V. L. Jones L. (2017). Social vulnerability to climate change: a review of concepts and evidence. *Reg. Environ. Change* 17 1651–1662. 10.1007/s10113-017-1105-9

[B120] Patrick R. Shaw A. Freeman A. Henderson-Wilson C. Lawson J. Davison M. (2019). Human wellbeing and the health of the environment: local indicators that balance the scales. *Soc. Indic. Res.* 146 651–667. 10.1007/s11205-019-02140-w

[B121] Pavlov V. Tracey K. (2012). The vagus nerve and the inflammatory reflex-linking immunity and metabolism. *Nat. Rev. Endocrinol.* 8 743–754. 10.1038/nrendo.2012.189 23169440 PMC4082307

[B122] Petersen E. Fiske A. P. Schubert T. W. (2019). The role of social relational emotions for human-nature connectedness. *Front. Psychol.* 10:2759. 10.3389/fpsyg.2019.02759 31920812 PMC6928140

[B123] Pickett K. E. Wilkinson R. G. (2015). Income inequality and health: a causal review. *Soc. Sci. Med.* 128 316–326. 10.1016/j.socscimed.2014.12.031 25577953

[B124] Porges S. W. (2011). *The Polyvagal Theory: Neurophysiological Foundations of Emotions, Attachment, Communication, and Self-regulation*, 1st Edn. New York, NY: W. W. Norton & Company.

[B125] Prati G. Albanesi C. Pietrantoni L. Airoldi L. (2016). Public perceptions of beach nourishment and conflict management strategies: a case study of Portonovo Bay in the Adriatic Italian Coast. *Land Use Policy* 50 422–428. 10.1016/j.landusepol.2015.06.033

[B126] Pritchard A. Richardson M. Sheffield D. McEwan K. (2019). The relationship between nature connectedness and eudaimonic well-being: a meta-analysis. *J. Happiness Stud.* 21 1145–1167. 10.1007/s10902-019-00118-6

[B127] Putnam R. D. (2000). *Bowling Alone.* New York, NY: Simon & Schuster.

[B128] Ray T. N. Franz S. A. Jarrett N. L. Pickett S. M. (2020). Nature enhanced meditation: effects on mindfulness, connectedness to nature, and pro-environmental behavior. *Environ. Behav.* 10.1177/0013916520952452 [Epub ahead of print].

[B129] Richardson M. Dobson J. Abson D. J. Lumber R. Hunt A. Young R. (2020a). Applying the pathways to nature connectedness at a societal scale: a leverage points perspective. *Ecosyst. People* 16 387–401. 10.1080/26395916.2020.1844296

[B130] Richardson M. McEwan K. Maratos F. Sheffield D. (2016). Joy and calm: how an evolutionary functional model of affect regulation informs positive emotions in nature. *Evol. Psychol. Sci.* 2 308–320. 10.1007/s40806-016-0065-5

[B131] Richardson M. Passmore H. Barbett L. Lumber R. Thomas R. Hunt A. (2020b). The green care code: how nature connectedness and simple activities help explain pro-nature conservation behaviours. *People Nat.* 2 821–839. 10.1002/pan3.10117

[B132] Ringov D. Zollo M. (2007). The impact of national culture on corporate social performance. *Corp. Gov. Int. J. Bus. Soc.* 7 476–485. 10.1108/14720700710820551

[B133] Ryan R. M. Deci E. L. (2001). On happiness and human potentials: a review of research on hedonic and eudaimonic well-being. *Annu. Rev. Psychol.* 52 141–166. 10.1146/annurev.psych.52.1.141 11148302

[B134] Ryff C. D. (2014). Psychological well-being revisited: advances in the science and practice of eudaimonia. *Psychother. Psychosom.* 83 10–28. 10.1159/000353263 24281296 PMC4241300

[B135] Ryff C. D. Love G. D. Urry H. L. Muller D. Rosenkranz M. A. Friedman E. M. (2006). Psychological well-being and ill-being: do they have distinct or mirrored biological correlates? *Psychother. Psychosom.* 75 85–95. 10.1159/000090892 16508343

[B136] Saeri A. K. Cruwys T. Barlow F. K. Stronge S. Sibley C. G. (2018). Social connectedness improves public mental health: Investigating bidirectional relationships in the New Zealand attitudes and values survey. *Aust. N. Z. J. Psychiatry* 52 365–374. 10.1177/0004867417723990 28803484

[B137] Santos H. C. Varnum M. E. W. Grossmann I. (2017). Global increases in individualism. *Psychol. Sci.* 28 1228–1239. 10.1177/0956797617700622 28703638

[B138] Saporito S. Casey D. (2015). Are there relationships among racial segregation, economic isolation, and proximity to green space? *Hum. Ecol. Rev.* 21 113–131.

[B139] Seligman M. (2012). *Flourish: A Visionary New Understanding of Happiness and Well-Being.* New York, NY: Atria Books.

[B140] Seligman M. (2017). PERMA and the building blocks of well-being. *J. Posit. Psychol.* 13 1–3. 10.1080/17439760.2020.1818813

[B141] Shanahan D. F. Astell–Burt T. Barber E. A. Brymer E. Cox D. T. C. Dean J. (2019). Nature–based interventions for improving health and wellbeing: the purpose, the people and the outcomes. *Sports* 7:141. 10.3390/sports7060141 31185675 PMC6628071

[B142] Sheeran P. (2002). Intention—behavior relations: a conceptual and empirical review. *Eur. Rev. Soc. Psychol.* 12 1–36. 10.1002/0470013478.ch1

[B143] Shiota M. N. Neufeld S. L. Yeung W. H. Moser S. E. Perea E. F. (2011). Feeling good: autonomic nervous system responding in five positive emotions. *Emotion* 11 1368–1378. 10.1037/a0024278 22142210

[B144] Skevington S. M. Böhnke J. R. (2018). How is subjective well-being related to quality of life? do we need two concepts and both measures? *Soc. Sci. Med.* 206 22–30. 10.1016/j.socscimed.2018.04.005 29680769

[B145] Stellar J. E. Gordon A. M. Piff P. K. Cordaro D. Anderson C. L. Bai Y. (2017). Self-transcendent emotions and their social functions: compassion, gratitude, and awe bind us to others through prosociality. *Emot. Rev.* 9 200–207. 10.1177/1754073916684557

[B146] Steptoe A. Deaton A. Stone A. A. (2015). Subjective wellbeing, health, and ageing. *Lancet* 385 640–648. 10.1016/s0140-6736(13)61489-0 25468152 PMC4339610

[B147] Steptoe A. Wardle J. Tsuda A. Tanaka Y. (2007). Depressive symptoms, socio-economic background, sense of control, and cultural factors in University students from 23 Countries. *Int. J. Behav. Med.* 14 97–107. 10.1007/bf03004175 17926438

[B148] Thayer J. Hansen A. L. Saus-Rose E. Johnsen B. H. (2009). Heart rate variability, prefrontal neural function, and cognitive performance: the neurovisceral integration perspective on self-regulation, adaptation, and health. *Ann. Behav. Med.* 37 141–153. 10.1007/s12160-009-9101-z 19424767

[B149] Tracey K. J. (2002). The inflammatory reflex. *Nature* 420 853–859. 10.1038/nature01321 12490958

[B150] Tulip C. Fisher Z. Bankhead H. Wilkie L. Pridmore J. Gracey F. (2020). Building wellbeing in people with chronic conditions: a qualitative evaluation of an 8-week positive psychotherapy intervention for people living with an acquired brain injury. *Front. Psychol.* 11:66. 10.3389/fpsyg.2020.00066 32082221 PMC7006056

[B151] Twenge J. M. (2013). Overwhelming evidence for generation me. *Emerg. Adulthood* 1 21–26. 10.1177/2167696812468112

[B152] Uchida Y. Oishi S. (2016). The happiness of individuals and the collective. *Jpn. Psychol. Res.* 58 125–141. 10.1111/jpr.12103

[B153] Umberson D. Montez J. K. (2010). Social relationships and health: a flashpoint for health policy. *J. Health Soc. Behav.* 51 (Suppl. 1) S54–S66. 20943583 10.1177/0022146510383501 PMC3150158

[B154] Uphoff E. P. Pickett K. E. Cabieses B. Small N. Wright J. (2013). A systematic review of the relationships between social capital and socioeconomic inequalities in health: a contribution to understanding the psychosocial pathway of health inequalities. *Int. J. Equity Health* 12:54. 10.1186/1475-9276-12-54 23870068 PMC3726325

[B155] Veitch J. Ball K. Crawford D. Abbott G. R. Salmon J. (2012). Park improvements and park activity a natural experiment. *Am. J. Prev. Med.* 42 616–619. 10.1016/j.amepre.2012.02.015 22608379

[B156] Venhoeven L. A. Bolderdijk J. W. Steg L. (2013). Explaining the paradox: how pro-environmental behaviour can both thwart and foster well-being. *Sustainability* 5 1372–1386. 10.3390/su5041372

[B157] Venhoeven L. A. Bolderdijk J. W. Steg L. (2016). Why acting environmentally-friendly feels good: exploring the role of self-image. *Front. Psychol.* 7:1846. 10.3389/fpsyg.2016.01846 27933017 PMC5121119

[B158] Verdugo V. C. (2012). The positive psychology of sustainability. *Environ. Dev. Sustain.* 14 651–666. 10.1007/s10668-012-9346-8

[B159] Wendelboe-Nelson C. Kelly S. Kennedy M. Cherrie J. W. A. (2019). Scoping review of mapping research on green space and associated mental health benefits. *Int. J. Environ. Res. Public Health* 16:2081. 10.3390/ijerph16122081 31212860 PMC6616579

[B160] Werner G. G. Ford B. Q. Mauss I. B. Schabus M. Blechert J. Wilhelm F. H. (2015). High cardiac vagal control is related to better subjective and objective sleep quality. *Biol. Psychol.* 106 79–85. 10.1016/j.biopsycho.2015.02.004 25709072 PMC4364614

[B161] Westerhof G. J. Keyes C. L. M. (2010). Mental illness and mental health: the two continua model across the lifespan. *J. Adult Dev.* 17 110–119. 10.1007/s10804-009-9082-y 20502508 PMC2866965

[B162] Whear R. Coon J. T. Bethel A. Abbott R. Stein K. Garside R. (2014). What is the impact of using outdoor spaces such as gardens on the physical and mental well-being of those with dementia? A systematic review of quantitative and qualitative evidence. *J. Am. Med. Dir. Assoc.* 15 697–705. 10.1016/j.jamda.2014.05.013 25037168

[B163] White M. Alcock I. Grellier J. Wheeler B. Hartig T. Warber S. (2019). Spending at least 120 minutes a week in nature is associated with good health and wellbeing. *Sci. Rep.* 9:7730. 31197192 10.1038/s41598-019-44097-3 PMC6565732

[B164] Wilker E. H. Wu C.-D. McNeely E. Mostofsky E. Spengler J. Wellenius G. A. (2014). Green space and mortality following ischemic stroke. *Environ. Res.* 133 42–48. 10.1016/j.envres.2014.05.005 24906067 PMC4151551

[B165] Wilkie L. Arroyo P. Conibeer H. Kemp A. H. Fisher Z. (2021). The impact of psycho-social interventions on the wellbeing of individuals with acquired brain injury during the COVID-19 pandemic. *Front. Psychol.* 12:648286. 10.3389/fpsyg.2021.648286 33841287 PMC8027334

[B166] Wilkinson R. G. Pickett K. (2010). *The Spirit Level: Why Greater Equality Makes Societies Stronger.* New York: Bloomsbury Press.

[B167] Wilkinson R. Pickett K. (2019). *The Inner Level How More Equal Societies Reduce Stress, Restore Sanity and Improve Everyone’s Well-being.* London: Penguin Books. 10.3399/bjgp19X705377 PMC6715479 31467020

[B168] Williams D. (2006). On and off the ’net: scales for social capital in an online era. *J. Comput. Mediat. Commun.* 11 593–628. 10.1111/j.1083-6101.2006.00029.x

[B169] Williams D. P. Cash C. Rankin C. Bernardi A. Koenig J. Thayer J. (2015). Resting heart rate variability predicts self-reported difficulties in emotion regulation: a focus on different facets of emotion regulation. *Front. Psychol.* 6:261. 10.3389/fpsyg.2015.00261 25806017 PMC4354240

[B170] Williams P. G. Cribbet M. R. Tinajero R. Rau H. K. Thayer J. F. Suchy Y. (2019). The association between individual differences in executive functioning and resting high-frequency heart rate variability. *Biol. Psychol.* 148:107772. 10.1016/j.biopsycho.2019.107772 31577925

[B171] Wong P. T. P. (2010). Meaning therapy: an integrative and positive existential psychotherapy. *J. Contemp. Psychother.* 40 85–93. 10.1007/s10879-009-9132-6

[B172] Wong P. T. P. (2012). “Toward a dual-systems model of what makes life worth living,” in *The Human Quest For Meaning: Theories, Research, And Applications*, ed. Wong P. T. P. (New York, NY: Routledge), 3–22.

[B173] Wong P. T. P. (2013). “Positive psychology,” in *Encyclopedia of Cross-Cultural Psychology*, ed. Keith K. (Oxford: Wiley Blackwell), 1021–1026.

[B174] Wong P. T. P. (2019). Second wave positive psychology’s (PP 2.0) contribution to counselling psychology. *Couns. Psychol. Q.* 32 1–10. 10.1080/09515070.2012.727670

[B175] Wright S. D. Wadsworth A. M. (2014). Gray and green revisited: a multidisciplinary perspective of gardens, gardening, and the aging process. *J. Aging Res.* 2014 1–13. 10.1155/2014/283682 24734179 PMC3964736

[B176] Yakushko O. (2019). Scientific Pollyannaism, From Inquisition to Positive Psychology, Palgrave Macmillan.

[B177] Yamakawa K. Rajendran P. S. Takamiya T. Yagishita D. So E. L. Mahajan A. (2015). Vagal nerve stimulation activates vagal afferent fibers that reduce cardiac efferent parasympathetic effects. *Am. J. Physiol. Heart C* 309 H1579–H1590. 10.1152/ajpheart.00558.2015 PMC4666973 26371172

[B178] Yang X.-F. Immordino-Yang M. H. (2017). Culture and cardiac vagal tone independently influence emotional expressiveness. *Cult Brain* 5 36–49. 10.1007/s40167-017-0048-9

[B179] Young H. A. Benton D. (2018). Heart-rate variability: a biomarker to study the influence of nutrition on physiological and psychological health? *Behav. Pharmacol.* 29 140–151. 10.1097/fbp.0000000000000383 29543648 PMC5882295

[B180] Zilioli S. Slatcher R. B. Ong A. D. Gruenewald T. L. (2015). Purpose in life predicts allostatic load ten years later. *J. Psychosom. Res.* 79 451–457. 10.1016/j.jpsychores.2015.09.013 26526322 PMC4684637

[B181] Zulfiqar U. Jurivich D. A. Gao W. Singer D. H. (2010). Relation of high heart rate variability to healthy longevity. *Am. J. Cardiol.* 105 1181–1185. 10.1016/j.amjcard.2009.12.022 20381674

